# Supplementary material for: Understanding influences of care-seeking behaviours for diarrhoeal illnesses: a qualitative meta-synthesis
Source: BMJ Open. 2026 Mar 4;16(3):e109903. doi: 10.1136/bmjopen-2025-109903 (PMC12970062; doi:10.1136/bmjopen-2025-109903)
Supplement: online supplemental file 1 [file bmjopen-16-3-s001.docx]

**Table S1.  Thematic Codebook.**

| **Parent Code** | **Child Code** | **Description** |
| --- | --- | --- |
| **Healthcare Providers Visited** | *Parent Code Description* | Types of healthcare providers visited |
|  | Health facility provider | When patients or caretakers go to see (OR wanted to go and see) a provider located at a health facility for care for diarrhea |
|  | CHW | When patients or caretakers go to see or request care from (OR wanted to go and see) a community health workers for diarrhea |
|  | Pharmacy | When patients or caretakers go to (OR wanted to go to) a pharmacy for diarrhea |
|  | OTC | When patients or caretakers go to (OR wanted to go to) a store for OTC medications  for diarrhea |
|  | Traditional Healer | When patients or caretakers go to see (OR wanted to go and see) a non-licensed community provider for diarrhea |
|  | Other | Seeing (OR wanting to go and see) other types of HCWs for diarrhea |
| **Clinical Factors Influencing Care-Seeking** | *Parent Code Description* | These codes capture how perceptions of the symptoms, severity, duration, origin of the diarrhea episode influence care-seeking behaviors/decisions |
|  | Symptoms | Refers to the specific clinical signs reported by the caregiver/experienced by the patient with diarrhea influencing care-seeking. |
|  | Illness duration | Refers to the length of time the individual with diarrhea has been experiencing symptoms influencing care-seeking. |
|  | Perceived severity | Refers to the caregiver or patient's subjective assessment of the seriousness/intensity of the diarrhea episode influencing care-seeking. |
|  | Perceived etiology | Refers to the caregiver or patient's understanding of the cause of the diarrhea episode influencing care-seeking. |
|  | Other | Refers to perceptions of other clinical factors influencing care-seeking. |
| **External Motivations Influencing Care-Seeking** | *Parent Code Description* | These codes identify external factors that push individuals towards care-seeking |
|  | Community | Use when non household or non family members are part of the decision making process for what care-seeking steps to take for diarrhea. |
|  | Family | Use when household or close family members advise or are part of the decision making process for what care-seeking steps to take for diarrhea. |
|  | Media/Technology | Use when messaging from the media or sources of technology influence the decision making process or care-seeking for diarrhea. |
|  | Other |  |
| **Internal or Caretaker Motivations Influencing Care-Seeking** | *Parent Code Description* | These codes focus on the internal thoughts and feelings of the caretakers or patients that motivate them to seek care for the diarrheal illness. |
|  | Perceived benefit | This code captures the individual's perception of the potential positive outcomes from seeking care for diarrhea. |
|  | Insecurity | Refers to a state of uncertainty, helplessness, and lack of confidence experienced by a caregiver or patient when dealing with diarrhea. |
|  | Confidence | Refers to a state of assurance and preparedness experienced by a caregiver or patient when dealing with diarrhea. This code highlights both the knowledge base (awareness, familiarity) and the emotional state (preparedness, trust) associated with confidence in managing diarrhea. |
|  | Other |  |
| **Structural and Logistic Factors Influencing Care-Seeking** | *Parent Code Description* | These codes identify practical and systemic factors that influence decision making about care-seeking, or hinder or facilitate care-seeking |
|  | Economic Factors | How or if financial capacity or limitations influence the decision to seek care for diarrhea. These can be presented as facilitators or barriers. |
|  | Access | How or if issues related to the availability and accessibility of healthcare services that impact care-seeking for diarrhea. These can be presented as facilitators or barriers. |
|  | Logistic Factors | How or if issues related to the logistics of care-seeking, beyond broader structural/systemic barriers or facilitators, impact care-seeking for diarrhea. These can be presented as facilitators or barriers. |
|  | Other |  |

Coding framework developed and applied in Atlas.ti during the initial coding phase. Parent codes represent anticipated broad thematic categories, while child code captures more specific sub-concepts.
