# Supplementary material for: Understanding influences of care-seeking behaviours for diarrhoeal illnesses: a qualitative meta-synthesis
Source: BMJ Open. 2026 Mar 4;16(3):e109903. doi: 10.1136/bmjopen-2025-109903 (PMC12970062; doi:10.1136/bmjopen-2025-109903)
Supplement: online supplemental file 2 [file bmjopen-16-3-s002.docx]

## **Table S2. Summary of included studies.** Each study included in the meta-synthesis, including reference number corresponding to citation in the main paper, study title, country and year study was conducted in, data collection methods used, and sample characteristics.

| No. | Study Title | Country | Year Published | Data Collection Methods | Sample Characteristics^1^ |
| --- | --- | --- | --- | --- | --- |
| [50] | Exploring health care seeking knowledge, perceptions and practices for childhood diarrhea and pneumonia and their context in a rural Pakistani community | Pakistan | 2018 | Focus group discussions, in-depth interviews, and narrative interviews | 45 participants (45/45 female)  20 narrative interviews (13/20 female) |
| [56] | Pediatric acute gastroenteritis: Understanding caregivers’ experiences and information needs | Canada | 2017 | Semi-structured, qualitative interviews | 15 participants (12/15 female) |
| [22] | Health seeking behaviour of mothers of under-five-year-old children in the slum communities of Nairobi, Kenya | Kenya | 2006 | In-depth interviews | 28 participants (28/28 female) |
| [23] | Mothers’ beliefs and barriers about childhood diarrhea and its management in Morang district, Nepal | Nepal | 2012 | Focus group discussions and in-depth interviews | 12 FGD participants (12/12 female), 8 IDIs (8/8 female) |
| [37] | Symptom-specific care-seeking behavior for sick neonates among urban poor in Lucknow, Northern India | India | 2008 | Focus group discussions | 43 participants (43/43 female) |
| [65] | Local barriers and solutions to improve care-seeking for childhood pneumonia, diarrhoea and malaria in Kenya, Nigeria and Niger: A qualitative study | Kenya, Nigeria, Niger | 2014 | In-depth interviews, focus group discussions | 65 IDIs, 91 FGD participants |
| [38] | Examining the use of oral rehydration salts and other oral rehydration therapy for childhood diarrhea in Kenya | Kenya | 2011 | Semi-structured group discussions, in-depth interviews | 45 participants |
| [54] | Unraveling diarrheal disease knowledge, understanding, and management practices among climate change vulnerable coastal communities in Ghana | Ghana | 2024 | Key informant interview, sex stratified focus group discussions | 67 FDG participants (35/67 female) |
| [44] | Mothers’ perceptions about their children’s acute diarrhea in the Quito suburbs | Ecuador | 2000 | Semi-structured interviews | 48 interviews (48/48 female) |
| [55] | “We don’t use the same ways to treat the illness:” A qualitative study of heterogeneity in health-seeking behaviour for acute gastrointestinal illness among the Ugandan Batwa | Uganda | 2022 | Focus group discussions, semi-structured interviews | 63 FGD participants (38/63 female), 11 interviews |
| [39] | A mother’s choice: A qualitative study of mothers’ health seeking behaviour for their children with acute diarrhoea | South Africa | 2016 | Semi-structured key informant interviews, focus group discussions | 32 FGD participants (32/32 female),  11 key informants |
| [63] | Healthcare seeking for diarrhoea, malaria and pneumonia among children in four poor rural districts in Sierra Leone in the context of free health care: Results of a cross-sectional survey | Sierra Leone | 2013 | Focus group discussion, in-depth interviews, follow-up interviews | 36 FGDs and 64 IDIs, including 15 follow-up interviews |
| [24] | Understanding factors influencing care seeking for sick children in Ebonyi and Kogi States, Nigeria | Nigeria | 2020 | In-depth interviews, focus group discussions | 60 IDIs,  48-60 FGD participants |
| [46] | Self-care as a response to diarrhoea in rural Bangladesh: Empowered choice or enforced adoption? Soc Sci Med, 63(10), 2686–2697 | Bangladesh | 2006 | Questionnaire, semi-structured interviews and illness narratives, focus group discussions | 208 participants (115/208 female) |
| [31] | Home management of childhood diarrhoea in southern Mali—Implications for the introduction of zinc treatment | Mali | 2007 | Illness narratives, semi-structured interviews | 14 illness narratives,  42 interviews |
| [32] | Ni-Vanuatu health-seeking practices for general health and childhood diarrheal illness: Results from a qualitative methods study | Vanuatu | 2015 | Group and individual interviews | 29 participants |
| [33] | “So they believe that if the baby is sick you must give drugs…” The importance of medicines in health-seeking behaviour for childhood illnesses in urban South Africa | South Africa | 2013 | Focus group discussions, in-depth interviews | 5 FGD (all female),  18 IDIs (18/18 female) |
| [25] | Home care of children with diarrhea in Bangui’s therapeutic landscape (Central African Republic) | Central African Republic | 2016 | In-depth interviews, focus group discussions, participant observations | 23 IDI (34 caregivers), 3 FDG, 37 participant observations, 7 health personnel, 2 unlicensed drug sellers |
| [47] | “My child can’t keep anything down!” Interviewing parents who bring their preschoolers to the emergency department for diarrhea, vomiting, and dehydration | Canada | 2010 | In-depth interviews | 10 families |
| [26] | Water quality and waterborne disease in the Niger River Inland Delta, Mali: A study of local knowledge and response | Mali | 2011 | Semi-structured interviews | 60 interviews (60/60 female) |
| [48] | Lived experience of acute gastrointestinal illness in Rigolet, Nunatsiavut: “Just suffer through it” | Canada | 2015 | In-depth interviews | 9 interviews (6/9 female) |
| [62] | Web-Based Knowledge Translation Tool About Pediatric Acute Gastroenteritis for Parents: Pilot Randomized Controlled Trial | Canada | 2023 | Semi-structured qualitative interview | 12 interviews |
| [60] | Recognizing childhood illnesses and their traditional explanations: Exploring options for care-seeking interventions in the context of the IMCI strategy in rural Ghana | Ghana | 2003 | In-depth interviews, semi-structured interviews | 58 IDIs, 60 semi-structured interviews |
| [52] | Listening to Caregivers: Narratives of Health Seeking for Children Under Five with Pneumonia and Diarrhea: Insights from the NIGRAAN Trial in Pakistan | 2023 | Pakistan | In-depth interviews | 20 interviews (13/20 female) |
| [57] | Healthcare Use for Diarrhoea and Dysentery in Actual and Hypothetical Cases, Nha Trang, Viet Nam | Vietnam | 2004 | Semi-structured interviews included the use of an interview guide and vignettes, and case studies | 109 interviews (63/109 female)  14 case studies (12/14 female) |
| [58] | Role of rotavirus vaccine in reducing diarrheal episodes in infants visiting private primary health care clinics in Karachi, Pakistan: A mixed-methods study. | Pakistan | 2024 | In-depth interviews, focus group discussions with mothers | 3 IDIs, 18 FGD participants (18/18 female) |
| [30] | Local Beliefs about Childhood Diarrhoea: Importance for Healthcare and Research | South Africa | 2004 | In-depth interviews, focus group discussions, card sorting exercises, case histories | 16 interviews (9/16 female), 5 card sorting participants, 13  case histories (13/13 female),  6 focus group participants |
| [34] | Strengthening nutrition services within integrated community case management (iCCM) of childhood illnesses in the Democratic Republic of Congo: Evidence to guide implementation | Democratic Republic of the Congo | 2019 | In-depth interviews, focus group discussions | 127 IDIs, 56 FGD participants |
| [64] | Drug use and self-medication among children with respiratory illness or diarrhea in a rural district in Vietnam: A qualitative study | Vietnam | 2011 | In-depth interviews, focus group discussions | 28 focus group participants (28/28 female) |
| [40] | Local perceptions of cholera and anticipated vaccine acceptance in Katanga province, Democratic Republic of Congo | Democratic Republic of the Congo | 2013 | In-depth interviews, focus group discussions, informal conversation, & semi-structured interview | 12 IDIs (6/12 female), 40 FGD participants (20/40 female), 360 interviewees (181/360 female) |
| [41] | Cultural influences behind cholera transmission in the Far North Region, Republic of Cameroon: A field experience and implications for operational level planning of interventions | Cameroon | 2017 | Key informant interviews, focus group discussions, guided household discussions | 90-120 FGD participants, 7 households |
| [29] | Treatment of Childhood Diarrhoea in Nigeria: Need for Adaptation of Health Policy and Programmes to Cultural Norms | Nigeria | 2000 | Two sex stratified focus group discussions, 2 mixed sex focus group discussions | 32-48 participants |
| [49] | Childhood diarrhoea in a district in northeast Thailand: Incidence and treatment choices | Thailand | 2006 | Focus group discussions, in-depth interviews, surveys | 3 focus groups, 121 IDIs |
| [27] | Elder authority and the situational diagnosis of diarrheal disease as normal infant development in northeast Thailand | Thailand | 2009 | Semi-structured, open-ended interviews | 64 interviews |
| [51] | Predictors of diarrheal mortality and patterns of caregiver health seeking behavior in in Karachi, Pakistan | Pakistan | 2016 | Focus group discussions | 29 participants (29/29 female) |
| [35] | Perspectives on child diarrhoea management and health service use among ethnic minority caregivers in Vietnam | Vietnam | 2011 | Semi-structured in-depth interviews, observations, focus group discussions | 43 interviews, 3 FGDs (0/24 female) |
| [66] | Environmental determinants of E. coli, link with the diarrheal diseases, and indication of vulnerability criteria in tropical West Africa (Kapore, Burkina Faso) | Burkina Faso | 2021 | Semi-structured interviews | 31 interviews |
| [36] | Navigating multiple options and social relationships in plural health systems: A qualitative study exploring healthcare seeking for sick children in Sierra Leone | Sierra Leone | 2014 | In-depth interviews, focus group discussions | 36 focus group participants (24/36 female) + 1 follow-up,  68 IDIs (68/68 female) + 2 follow-up |
| [28] | Influences on healthcare-seeking during final illnesses of infants in under-resourced South African settings | South Africa | 2011 | Semi-structured interviews, key-informant in-depth interviews | 39 interviews (39/39 female), 19 key informant IDIs |
| [42] | Access to integrated community case management of childhood illnesses services in rural Ethiopia: A qualitative study of the perspectives and experiences of caregivers | Ethiopia | 2016 | In-depth interviews, focus group discussions | 78 IDIs,  132 FGD participants (132/132 female) |
| [59] | Operationalising integrated community case management of childhood illnesses by community health workers in rural Haryana | India | 2018 | In-depth interviews, focus group discussions | 37 focus groups participants (37/37 female),  43 IDIs (43/43 female) |
| [61] | Circumstances of post-neonatal deaths in Ceara, Northeast Brazil: Mothers’ health care-seeking behaviors during their infants’ fatal illness | Brazil | 2000 | Semi-structured interviews | 127 interviews (127/127 female) |
| [43] | Treatment-seeking and recovery among young undernourished children post-hospital discharge in Bangladesh: A qualitative study | Bangladesh | 2022 | Repeat in-depth interviews, key informant interviews, focus group discussions | 73 repeat IDIs  15 Key informant interviews  18 FGD participants |
| [67] | Knowledge of, attitudes toward, and preventive practices relating to cholera and oral cholera vaccine among urban high-risk groups: Findings of a cross-sectional study in Dhaka, Bangladesh | Bangladesh | 2013 | In-depth interviews | 30 interviews (15/30 female) |
| [21] | “This diarrhoea is not a disease …” local illness concepts and their effects on mothers’ health seeking behaviour: A qualitative study, Shuhair, Yemen | Yemen | 2014 | Focus group discussions | 31 FGD participants (31/31 female) |
| [45] | A qualitative study of community perceptions about childhood diarrhea and its management in Assosa District, West Ethiopia | Ethiopia | 2014 | In-depth interviews, focus group discussions | 72 participants (60/72 female) |
| [53] | Comparing reports of health-seeking behavior from the integrated illness history and a standard child morbidity survey | Egypt | 2008 | Interviews that combined techniques from in-depth interviewing and standard survey interviewing | 137 participants |

^1^Information on sex of respondents provided only when available in text.
