# Supplementary material for: Understanding influences of care-seeking behaviours for diarrhoeal illnesses: a qualitative meta-synthesis
Source: BMJ Open. 2026 Mar 4;16(3):e109903. doi: 10.1136/bmjopen-2025-109903 (PMC12970062; doi:10.1136/bmjopen-2025-109903)
Supplement: Supplementary file 4 [file bmjopen-2025-109903.reviewer_comments.pdf]

PEER REVIEW HISTORY

BMJ Open publishes all reviews undertaken for accepted manuscripts. Reviewers are asked to complete a checklist review form and are provided with free text boxes to elaborate on their assessment. These free text comments are reproduced below.

ARTICLE DETAILS

Title (Provisional)

Understanding influences of care-seeking behaviors for diarrheal illnesses: A qualitative meta-synthesis

Authors

Miller, Marissa H; Hilbert, Skye M; Rosser, Erica; Sinko, Laura; Lee, Elizabeth C; Wiens, Kirsten E.

VERSION 1 - REVIEW

**Reviewer**	**1**
**Name**	**Giannouli, Vaitsa**
**Affiliation**	**Aristotle University of Thessaloniki**
**Date**	**04-Nov-2025**
**COI**	**None**

This is a very interesting article-qualitative meta-synthesis. Some points for a major revision are below:

Authors should discuss and compare their findings in the Results section with findings even if these findings concern other disorders and cultural contexts (for example the importance of atitudes towards endocrine diseases in Greece seems to play : https://link.springer.com/article/10.1007/s12020-016-1164-6).

Furthermore, regarding the findings over parents and their sick child, findings from other countries seem to support the importance of examining negative emotions of adult caregivers/parents on the access and treatment of their child as well as the relationship with the doctor-healthcare system (for a relevant article on this point to use: https://psyct.swu.bg/index.php/psyct/article/view/86)

In the Methods section, authors need to justify why they chose Atlas.ti for the analyses.

Adding figures to the Results section with the main findings would be useful instead of large tables.Furthermore, for additional tables with required information to include, please check the appendices of the article (and if you wish mention it as a reference in the Methods section for the applied methodology: https://www.mdpi.com/2227-9032/12/8/828) and add

relevant information for your article as well. Overall, this article deals with a very interesting and little-investigated topic!

**Reviewer**	**2**
**Name**	**Scallan Walter, Elaine**
**Affiliation**	**University of Colorado Denver - Anschutz Medical Campus**
**Date**	**11-Nov-2025**
**COI**	**None**

I really enjoyed reading this article on “Understanding influences of care-seeking behaviors for diarrheal illnesses: A qualitative meta-synthesis”. This is an important topic. The paper is well written and clear; the methods are well described.

Most identified articles were from lower income countries, though the authors sought to include studies from all over the world (which is a plus). However, I think the scope of the review could be made clearer in the abstract and introduction. I spent some time trying to figure this out at the beginning.

I also recommend that the authors list the countries with included studies in the text of the results rather than using broad geographic regions (e.g., north and south America) and referring the reader to Table 1. It is notable that four of the six articles for the Americas are from Canada, for example. There does not seem to be an excessive number of countries, and some have multiple articles/studies. I think it is worth naming them.

Also, those lines, the Canadian studies were often used for quotes. Most of the quotes from theme two are from Cananda, which left my wondering if there with differences between HIC and LMICs – but there are other studies listed under theme 2 in Table 2. Can the authors review each section to ensure that a variety of HIC and LMICs are used for quotes, unless there are some differences (in which case, it would be great to point that out). To that point, are there any differences between HIC and LMICs? Can that be mentioned in the discussion?

Page 4. End of the first paragraph. In addition to burden and resource allocation, the reporting of cases to health surveillance systems is vital for assessing disease trends and detection disease outbreaks. This should also be mentioned.

VERSION 1 - AUTHOR RESPONSE

Reviewer #1

1. This is a very interesting article-qualitative meta-synthesis. Some points for a major revision are below: Authors should discuss and compare their findings in the Results

section with findings even if these findings concern other disorders and cultural contexts (for example the importance of attitudes towards endocrine diseases in Greece seems to play : https://link.springer.com/article/10.1007/s12020-016-1164-6). Furthermore, regarding the findings over parents and their sick child, findings from other countries seem to support the importance of examining negative emotions of adult caregivers/parents on the access and treatment of their child as well as the relationship with the doctor-healthcare system (for a relevant article on this point to use: https://psyct.swu.bg/index.php/psyct/article/view/86)

Response: Thank you for this suggestion, and for sharing those references. We agree condition comparisons can offer interesting insights. However, given how varied and contextual care seeking was in each situation we came across, extending the analysis to other conditions falls outside of the intended scope of this review.

2. In the Methods section, authors need to justify why they chose Atlas.ti for the analyses.

   Response: We have included this justification in the methods, please see lines 158-162.

3. Adding figures to the Results section with the main findings would be useful instead of large tables. Furthermore, for additional tables with required information to include, please check the appendices of the article (and if you wish mention it as a reference in the Methods section for the applied methodology: https://www.mdpi.com/2227-9032/12/8/828) and add relevant information for your article as well. Overall, this article deals with a very interesting and little-investigated topic!

   Response: We appreciate this recommendation and reviewed the suggested structure. At this stage, we have retained the current tables, as they provide the most comprehensive and clear presentation of the synthesized findings for this review.

Reviewer #2

1. I really enjoyed reading this article on “Understanding influences of care-seeking behaviors for diarrheal illnesses: A qualitative meta-synthesis”. This is an important topic. The paper is well written and clear; the methods are well described.

   Response: Thank you, we really appreciate the kind words!

2. Most identified articles were from lower income countries, though the authors sought to include studies from all over the world (which is a plus). However, I think the scope of the review could be made clearer in the abstract and introduction. I spent some time trying to figure this out at the beginning.

   Response: Thank you for pointing this out. Our intention was to describe a globally inclusive scope, as we did not restrict inclusion to any certain areas. Most eligible studies originated from sub-Saharan Africa. We have made some revisions to the introduction and abstract to ensure this is state as clearly as possible.

3. I also recommend that the authors list the countries with included studies in the text of the results rather than using broad geographic regions (e.g., north and south America) and referring the reader to Table 1. It is notable that four of the six articles for the Americas are from Canada, for example. There does not seem to be an excessive number of countries, and some have multiple articles/studies. I think it is worth naming them.

   Response: We have revised the mentioned section to list the countries by name and have the reference for Table 1 included.

4. Also, those lines, the Canadian studies were often used for quotes. Most of the quotes from theme two are from Cananda, which left my wondering if there with differences between HIC and LMICs – but there are other studies listed under theme 2 in Table 2. Can the authors review each section to ensure that a variety of HIC and LMICs are used for quotes, unless there are some differences (in which case, it would be great to point that out). To that point, are there any differences between HIC and LMICs? Can that be mentioned in the discussion?

Response: Thank you for raising this point, as differences between HIC and LMIC were something of great interest to us while working on this project. We did not find any clear differences between the two. However, it is difficult for us to draw conclusions about this because we had a very limited number of HIC countries to compare LMIC with. We've described this in the limitations section.

The Canadian studies included in our review also provided a larger number of quotes discussing specific aspects of care seeking, more so than studies from other countries, which closely aligned with our presentation of the sub-themes linearly and is why they appear more frequently. However, in response to your comment, we have revised sections 2.1-3.2 to include more quotes or mentions from other locations to more accurately reflect the breadth of studies that touched on these themes.

5. Page 4. End of the first paragraph. In addition to burden and resource allocation, the reporting of cases to health surveillance systems is vital for assessing disease trends and detection disease outbreaks. This should also be mentioned.

   Response: Thank you, this is an important clarification. We have updated the paragraph to include that aspect as well, please see lines 76-77.

VERSION 2 - REVIEW

**Reviewer**	**2**
**Name**	**Scallan Walter, Elaine**
**Affiliation**	**University of Colorado Denver - Anschutz Medical Campus**
**Date**	**07-Jan-2026**
**COI**	

No additional comments. Nice article!
